# Supplementary material for: Differential gene expression elicited by ZIKV infection in trophoblasts from congenital Zika syndrome discordant twins
Source: PLoS Negl Trop Dis. 2020 Aug 3;14(8):e0008424. doi: 10.1371/journal.pntd.0008424 (PMC7425990; doi:10.1371/journal.pntd.0008424)
Supplement: S1 Fig — Immunostaining for (A) KRT7 and (B) CGB determined in three different replicates for each twin. No statistically significant differences among the different twins were observed (One-way ANOVA, Tukey's post-test, n = 3 replicates for each twin). (PDF) [file pntd.0008424.s001.pdf]

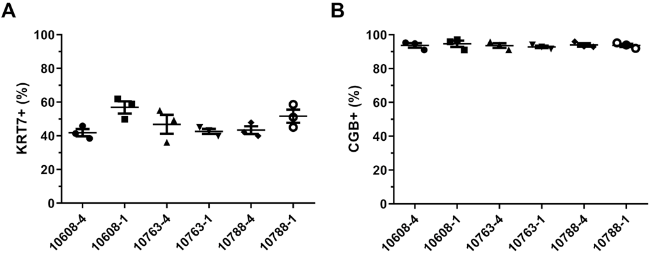

S1 Fig. Quantification of positive staining for trophoblast markers in hiPSC-derived trophoblast cell lines from each of the six twins. Immunostaining for (A) KRT7 and (B) CGB determined in three different replicates for each twin. No statistically significant differences among the different twins were observed (One-way ANOVA, Tukey's post-test, n = 3 replicates for each twin).
