# Supplementary figures and images for: Differential gene expression elicited by ZIKV infection in trophoblasts from congenital Zika syndrome discordant twins
Source: PLoS Negl Trop Dis. 2020 Aug 3;14(8):e0008424. doi: 10.1371/journal.pntd.0008424 (PMC7425990; doi:10.1371/journal.pntd.0008424)

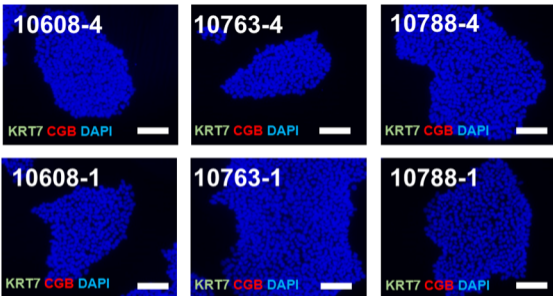

**S2 Fig. Undifferentiated iPSC cell lines from each of the six twins stained for KRT7 and CGB.**

Bar = 100  $\mu$ m.

Supplement: S2 Fig — Bar = 100 μm. (PDF) [file pntd.0008424.s002.pdf]
