# Supplementary material for: Differential gene expression elicited by ZIKV infection in trophoblasts from congenital Zika syndrome discordant twins
Source: PLoS Negl Trop Dis. 2020 Aug 3;14(8):e0008424. doi: 10.1371/journal.pntd.0008424 (PMC7425990; doi:10.1371/journal.pntd.0008424)
Supplement: S3 Fig — Expression measured by RT-qPCR of NANOG, a hiPSC marker, and of HCGA, HCGB and KRT7, three of the genes upregulated in the trophoblasts as compared with the hiPSCs. Twins from each family are represented with a different color: red, #10608 twins; blue, #10763 twins; green, #10788 twins. (mean ± SEM; n = 2 biological replicates, except for #10763 due to sample loss during culture) (PDF) [file pntd.0008424.s003.pdf]

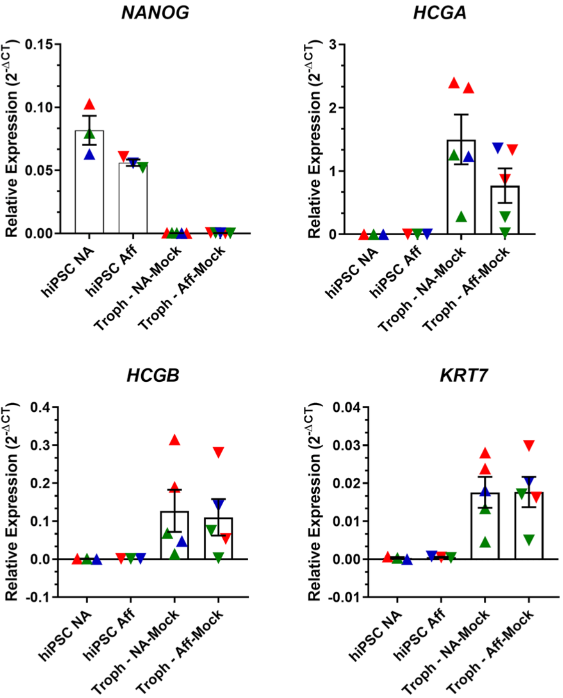

S3 Fig. Expression measured by RT-qPCR of marker genes of hiPSCs and trophoblasts. Expression measured by RT-qPCR of *NANOG*, a hiPSC marker, and of *HCGA*, *HCGB* and *KRT7*, three of the genes upregulated in the trophoblasts as compared with the hiPSCs. Twins from each family are represented with a different color: red, #10608 twins; blue, #10763 twins; green, #10788 twins. (mean ± SEM; n = 2 biological replicates, except for #10763 due to sample loss during culture)
