# Supplementary material for: Differential gene expression elicited by ZIKV infection in trophoblasts from congenital Zika syndrome discordant twins
Source: PLoS Negl Trop Dis. 2020 Aug 3;14(8):e0008424. doi: 10.1371/journal.pntd.0008424 (PMC7425990; doi:10.1371/journal.pntd.0008424)
Supplement: S4 Fig — Related to Fig 2. The bars represent expression levels (in TPM) of selected genes associated with cellular proliferation in hiPSCs from non-affected (light blue, hiPSC NA) or CZS-affected (dark blue, hiPSC Aff) twins, in the hiPSC-derived trophoblasts from non-affected twins’ mock (yellow, Troph–NA-Mock) or ZIKV-infected cells (orange, Troph–NA-MOI 0.3), and in the hiPSC-derived trophoblasts from CZS-affected twins’ mock (red, Troph–Aff-Mock) or ZIKV-infected cells (brown, Troph–Aff-MOI 0.3). The levels of expression of the genes were compared between non-affected (both non-infected and infected) trophoblasts and non-affected hiPSCs; and from CZS-affected (both non-infected and infected) trophoblasts and CZS-affected hiPSCs. Genes significantly down-regulated in trophoblast cells when compared with hiPSCs are shown (one-away ANOVA, p-value threshold was * 0.05, ** 0.01, *** 0.001 and **** 0.0001). Error bars show SEM. (PDF) [file pntd.0008424.s004.pdf]

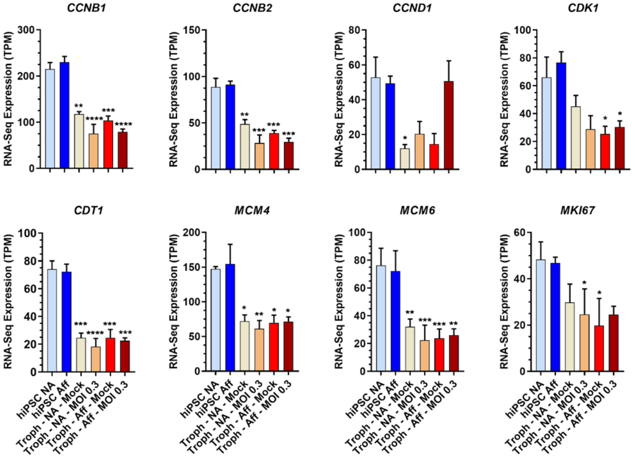

S4 Fig. Expression levels measured by RNA-Seq of proliferation-related genes in the hiPSCs and in the hiPSC-derived trophoblasts from non-affected or CZS-affected twins. Related to Figure 2. The bars represent expression levels (in TPM) of selected genes associated with cellular proliferation in hiPSCs from non-affected (light blue, hiPSC NA) or CZS-affected (dark blue, hiPSC Aff) twins, in the hiPSC-derived trophoblasts from non-affected twins' mock (yellow, Troph – NA-Mock) or ZIKV-infected cells (orange, Troph – NA-MOI 0.3), and in the hiPSC-derived trophoblasts from CZS-affected twins' mock (red, Troph – Aff-Mock) or ZIKV-infected cells (brown, Troph – Aff-MOI 0.3). The levels of expression of the genes were compared between non-affected (both non-infected and infected) trophoblasts and non-affected hiPSCs; and from CZS-affected (both non-infected and infected) trophoblasts and CZS-affected hiPSCs. Genes significantly down-regulated in trophoblast cells when compared with hiPSCs are shown (one-away ANOVA, p-value threshold was * 0.05, ** 0.01, *** 0.001 and **** 0.0001). Error bars show SEM.
