# Supplementary material for: Differential gene expression elicited by ZIKV infection in trophoblasts from congenital Zika syndrome discordant twins
Source: PLoS Negl Trop Dis. 2020 Aug 3;14(8):e0008424. doi: 10.1371/journal.pntd.0008424 (PMC7425990; doi:10.1371/journal.pntd.0008424)
Supplement: S7 Fig — Related to Fig 2. The bars represent expression levels (in TPM) of genes encoding interferon receptors in hiPSCs from non-affected (light blue, hiPSC NA) or CZS-affected (dark blue, hiPSC Aff) twins, in the hiPSC-derived trophoblasts from non-affected twins’ mock (yellow, Troph–NA-Mock) or ZIKV-infected cells (orange, Troph–NA-MOI 0.3), and in the hiPSC-derived trophoblasts from CZS-affected twins’ mock (red, Troph–Aff-Mock) or ZIKV-infected cells (brown, Troph–Aff-MOI 0.3). None of these genes was significantly differentially expressed in hiPSC-derived trophoblasts from CZS-affected twins when compared with hiPSC-derived trophoblasts from non-affected twins in pairwise comparisons (two-tailed t-test, equal variance). Error bars show SEM. (PDF) [file pntd.0008424.s007.pdf]

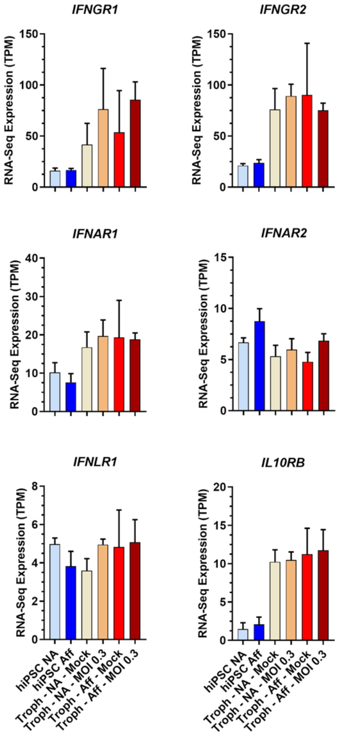

S7 Fig. Expression levels measured by RNA-Seq of genes encoding receptors for Type I, Type II, and Type III IFNs. Related to Figure 2. The bars represent expression levels (in TPM) of genes encoding interferon receptors in hiPSCs from non-affected (light blue, hiPSC NA) or CZS-affected (dark blue, hiPSC Aff) twins, in the hiPSC-derived trophoblasts from non-affected twins' mock (yellow, Troph – NA-Mock) or ZIKV-infected cells (orange, Troph – NA-MOI 0.3), and in the hiPSC-derived trophoblasts from CZS-affected twins' mock (red, Troph – Aff-Mock) or ZIKV-infected cells (brown, Troph – Aff-MOI 0.3). None of these genes was significantly differentially expressed in hiPSC-derived trophoblasts from CZS-affected twins when compared with hiPSC-derived trophoblasts from non-affected twins in pairwise comparisons (two-tailed t-test, equal variance). Error bars show SEM.
