# Supplementary material for: Differential gene expression elicited by ZIKV infection in trophoblasts from congenital Zika syndrome discordant twins
Source: PLoS Negl Trop Dis. 2020 Aug 3;14(8):e0008424. doi: 10.1371/journal.pntd.0008424 (PMC7425990; doi:10.1371/journal.pntd.0008424)
Supplement: S8 Fig — Related to Fig 4. Gene Ontology terms enrichment analysis of upregulated genes in hiPSC-derived trophoblasts from CZS-affected compared with non-affected twins after ZIKVBR in vitro infection. The major GO term categories, namely Biological Process and Molecular Function are separately represented in each panel. The size of the circles is proportional to the number of genes in each significantly enriched category, as indicated by the scale at right; the colors show the statistical significance of the enrichment, as indicated by the -log10 FDR values that appear in the color-coded scale at right. A GO enrichment significance cutoff of FDR ≤ 0.05 was used. (PDF) [file pntd.0008424.s008.pdf]

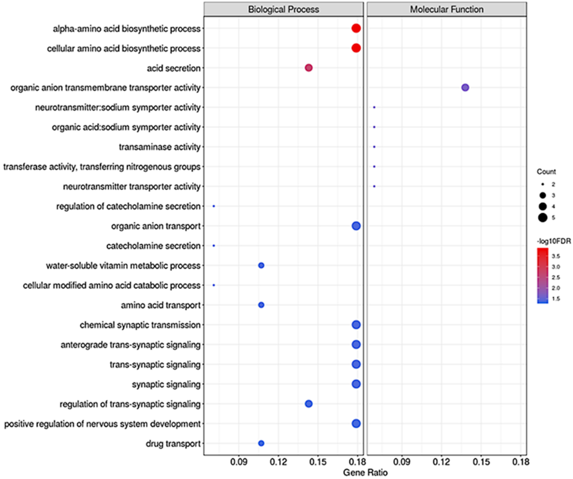

S8 Fig. Differential gene expression between hiPSC-derived trophoblast from CZS-affected and non-affected twins after ZIKV^{BR} infection. Related to Figure 4. Gene Ontology terms enrichment analysis of upregulated genes in hiPSC-derived trophoblasts from CZS-affected compared with non-affected twins after ZIKV^{BR} in vitro infection. The major GO term categories, namely Biological Process and Molecular Function are separately represented in each panel. The size of the circles is proportional to the number of genes in each significantly enriched category, as indicated by the scale at right; the colors show the statistical significance of the enrichment, as indicated by the -log10 FDR values that appear in the color-coded scale at right. A GO enrichment significance cutoff of FDR ≤ 0.05 was used.
