# Supplementary material for: Differential gene expression elicited by ZIKV infection in trophoblasts from congenital Zika syndrome discordant twins
Source: PLoS Negl Trop Dis. 2020 Aug 3;14(8):e0008424. doi: 10.1371/journal.pntd.0008424 (PMC7425990; doi:10.1371/journal.pntd.0008424)
Supplement: S9 Fig — Expression measured by RT-qPCR of COL3A1, ITGA1 and LGALS3, three of the genes downregulated after ZIKVBR infection in trophoblasts from CZS-affected (Aff) when compared with non-affected (NA) twins. Twins from each family are represented with a different color: red, #10608 twins; blue, #10763 twins; green, #10788 twins. Mean ± SEM is shown. (n = 2 biological replicates, except for #10763 due to sample loss during culture; One-tailed t-test, * p<0.05). (PDF) [file pntd.0008424.s009.pdf]

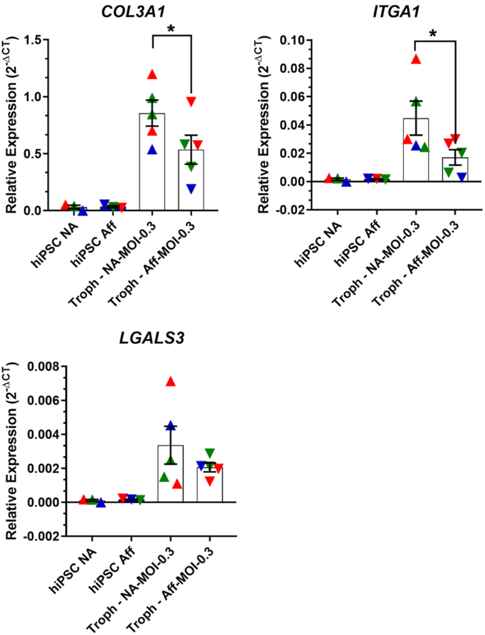

S9 Fig. Expression measured by RT-qPCR of genes found in the RNA-Seq analysis downregulated after ZIKV^{BR} infection in trophoblasts from CZS-affected when compared with non-affected twins. Expression measured by RT-qPCR of *COL3A1*, *ITGA1* and *LGALS3*, three of the genes downregulated after ZIKV^{BR} infection in trophoblasts from CZS-affected (Aff) when compared with non-affected (NA) twins. Twins from each family are represented with a different color: red, #10608 twins; blue, #10763 twins; green, #10788 twins. Mean ± SEM is shown. (n = 2 biological replicates, except for #10763 due to sample loss during culture; One-tailed t-test, * $p<0.05$).
